# Enhancing nanoparticle accumulation in two dimensional, three dimensional, and xenograft mouse cancer cell models in the presence of docetaxel

**DOI:** 10.1038/s41598-022-17752-5

**Published:** 2022-08-05

**Authors:** Kyle Bromma, Nancy Dos Santos, Ingrid Barta, Abraham Alexander, Wayne Beckham, Sunil Krishnan, Devika B. Chithrani

**Affiliations:** 1 Department of Physics and Astronomy, University of Victoria, Victoria, BC Canada; 2 British Columbia Cancer Research Institute, Vancouver, BC Canada; 3 Animal Care Services, University of British Columbia, Vancouver, BC Canada; 4 Department of Surgery, University of British Columbia, Vancouver, BC Canada; 5 British Columbia Cancer, Victoria, BC Canada; 6 Department of Radiation Oncology, Mayo Clinic, Jacksonville, Florida USA; 7 Centre for Advanced Materials and Related Technologies (CAMTEC), University of Victoria, Victoria, BC Canada; 8 Division of Medical Sciences, University of Victoria, Victoria, BC Canada

**Keywords:** Cancer models, Nanoparticles, Drug delivery, Nanotechnology in cancer

## Abstract

Recent clinical trials show docetaxel (DTX), given in conjunction with radiation therapy (RT) and androgen suppression, improves survival in high-risk prostate cancer. Addition of gold nanoparticles (GNPs) to this current DTX/RT protocol is expected to further improve therapeutic benefits remarkably. However, the foundation for the triple combination of RT, DTX, and GNPs must be elucidated to ensure quicker facilitation to the clinic. In this study, we explored the use of low concentrations of DTX combined with GNPs in two prostate cancer cell lines in a two-dimensional monolayer, a three-dimensional spheroid, and a mouse xenograft model. When used together, DTX and GNPs induced a nearly identical relative increase in uptake of gold in both the spheroid model and the mouse xenograft, which saw a 130% and 126% increase respectively after 24 h, showcasing the benefit of using spheroids as an in vitro model to better optimize in vivo experiments. Further, the benefits of using low concentrations of DTX combined with GNPs extended for over 72 h, allowing for less frequency in dosing when translating to the clinic. Overall, these results highlight the benefits of using DTX combined with GNPs and lays the groundwork for the translation of the triple combination of RT, GNPs, and DTX to the clinic.

## Introduction

Prostate cancer (PCa) is the leading cause of cancer in men worldwide (excluding non-melanoma skin cancer), at 14.1% of all new cases, and accounts for 7% of deaths due to cancer in males^1^. Management options for localized disease may include active surveillance, surgery, external beam radiation therapy (RT), or brachytherapy. High risk disease often requires multimodal management, with consideration given to the addition of systemic therapies such as androgen suppression or chemotherapy to improve outcome^2^. While many of these treatment options have seen some success, the quality of life for many of the patients following treatment can be reduced. Between active watching, prostatectomy, and external beam radiotherapy, radiotherapy had a larger negative side effect on sexual function as well as bowel function^3^. The introduction of advanced RT planning and delivery techniques (eg. IMRT, VMAT) as well as image guided RT has enabled dose escalation with reduced toxicity, improving outcome and better quality of life for patients^4–6^. However, urinary and gastrointestinal toxicity still occur in greater than 20% of patients 6 months after treatment and in greater than 45% of patients 3 years after treatment^7^. Improvements to deposited dose while reducing normal tissue toxicity is paramount for all RT-based treatment options, and nanotechnology is one option to achieve this.

High atomic number noble metal nanoparticles (NPs), such as gold nanoparticles (GNPs; Z = 79) offer many advantages as a model nanomaterial-based system. This can include benefits such as bio-inertness and biostability, while also being simple to functionalize with various moieties^8,9^. GNPs have previously been shown to act as an effective radiosensitizer in clinically relevant energy ranges^10–12^. Targeting of GNPs to tumors can selectively increase deposited dose, increasing the therapeutic index by reducing normal tissue toxicity^13,14^. Further, GNPs can be targeted to tumours, such as by the addition of the RGD peptide, which also improves the uptake of GNPs through increased endocytic effects^15,16^.

The addition of chemotherapy drugs has been explored for patients who have metastatic PCa^17^. For metastatic castration-resistant PCa, the taxane Docetaxel (DTX) is the first line option in therapy^18,19^. In the PEACE-1 study, combination of DTX with androgen deprivation therapy, abiraterone, and radiotherapy resulted in an improved overall survival and radiographic progression-free survival in metastatic castration-sensitive PCa^20^. Taxanes such as DTX are antimitotic agents that work by inhibiting division of cells through unregulated formation of microtubules^21^. This has the added benefit of blocking the cells’ ability to go through mitosis, synchronizing the cells in the G2/M phase of the cell cycle, the most radiosensitive phase of the cell cycle^22^. Thus, DTX can act as a radiosensitizer and has been used in multiple clinical trials^23–25^.

**Figure 1:**
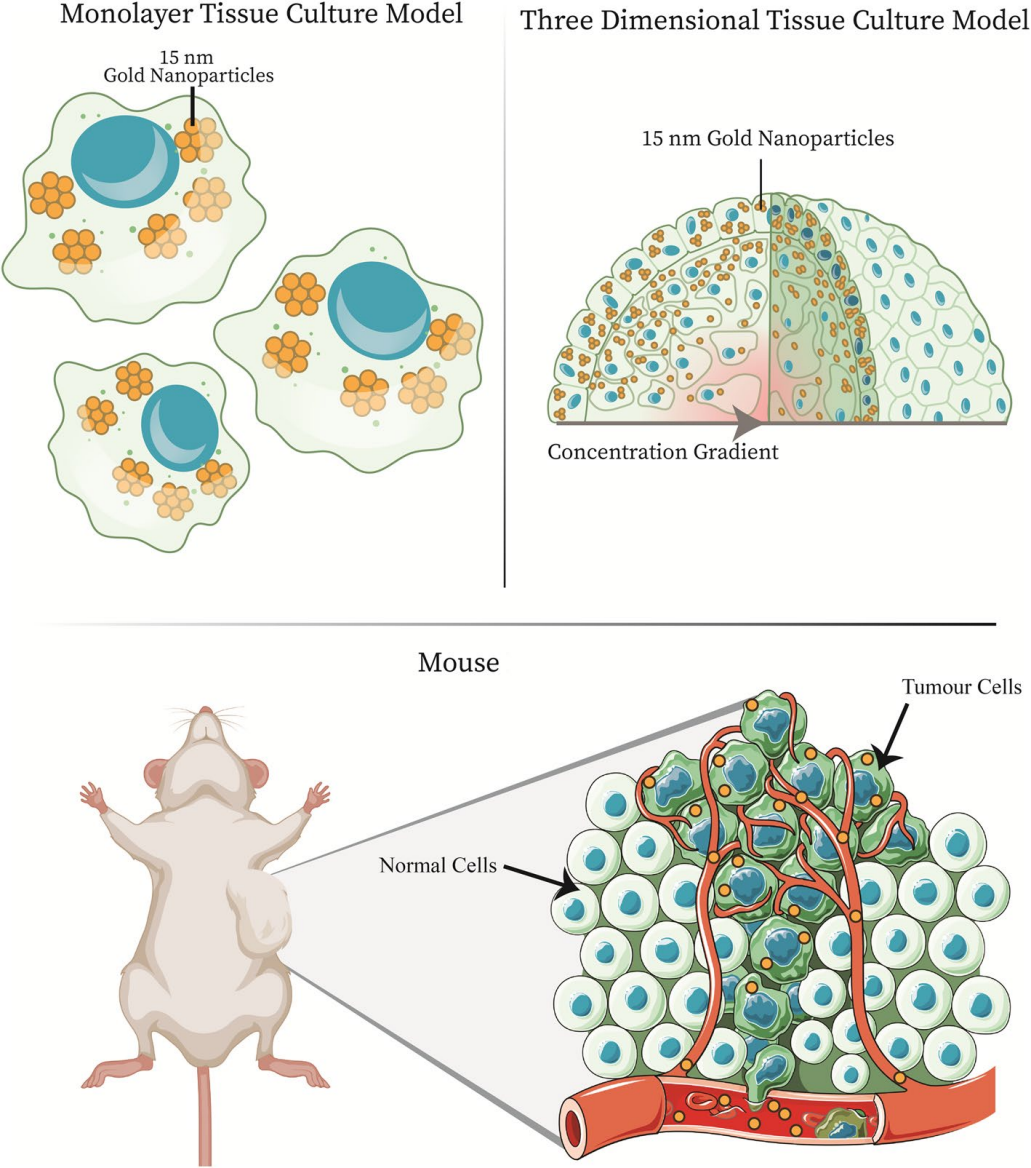
**Pre-clinical models for testing efficacy of gold nanoparticles.** There are three main models that can be utilized pre-clinically for measuring and optimizing the delivery of gold nanoparticles. First, we have the two-dimensional monolayer tissue culture model, which involves single cells. For further complexity, the introduction of a three-dimensional spheroid model allows for improved experimentation, as it better mimics the in-vivo environment, including the addition of an extracellular matrix. Finally, you have animal xenograft models, that allow for the most accurate testing before moving into clinical trials. In animal xenograft models, you have immune infiltrate, tumour vasculature, and a complete tumour microenvironment. Mouse in figure created with BioRender.com

As a result, the combination of low dose DTX and GNPs has been explored as an option for combined therapy in two-dimensional (2D) monolayers and in three-dimensional (3D) spheroids, like those found in Fig. 1^26,27^. It has been shown that non-toxic doses of DTX can increase the uptake of GNPs, and that the addition of DTX to a GNP treatment leads to a synergistic effect when combined with RT, in monolayer^28^. In a spheroid model, there is an introduction of a more complex environment, including an extracellular matrix which leads to a concentration gradient, which is similar to what occurs in vivo. However, the uptake of GNPs when combined with DTX has not been explored in vivo. This is an essential step before combined GNP + DTX + RT treatment can be explored in vivo. As Fig. 1 shows, the step towards an in vivo environment adds even more complexity, with introduced immune infiltrate, chaotic tumour vasculature, and a complete tumor microenvironment, including stromal cells like fibroblasts.

Thus, the work performed in this study involves the use of GNPs functionalized with polyethylene glycol (PEG) and a peptide containing the integrin binding domain RGD. The process of PEGylation forms a protective hydrating layer, which in turn hinders protein adsorption and clearance by macrophages, resulting in stability of the GNP in serum^29,30^. The addition of RGD is due to the increased expression of integrin dimers—i.e. αvβ3, αvβ5, and α5β1, which can recognize the RGD motif – in various cancers associated with poor patient outcomes, allowing for specific targeting^31^. These GNPs were then combined with low doses of DTX to compare the different gold nanoparticle uptake kinetics in a 2D monolayer model, 3D spheroid model, and in vivo mouse xenograft model, all with prostate cancer cell lines, as seen in Fig. 1. While a 2D monolayer model is a good basis for further experimentation, it does not properly simulate the complex heterogenous environment present in a tumor microenvironment. The introduction of a 3D spheroid model can allow for a more accurate approximation of the in vivo environment and accelerate the use of radiosensitizers like GNPs to the clinic. For the 2D model and 3D model, we use two prostate cancer cell lines, PC-3 and LNCaP, and for the mouse xenograft model, we used a subcutaneous tumour model with PC-3. By determining and comparing GNP uptake in all three models when combined with DTX, we can lay the foundation for future experiments that facilitate a quicker translation to the clinic.

## Results and discussion

### Characterization of gold nanoparticle complex

**Figure 2:**
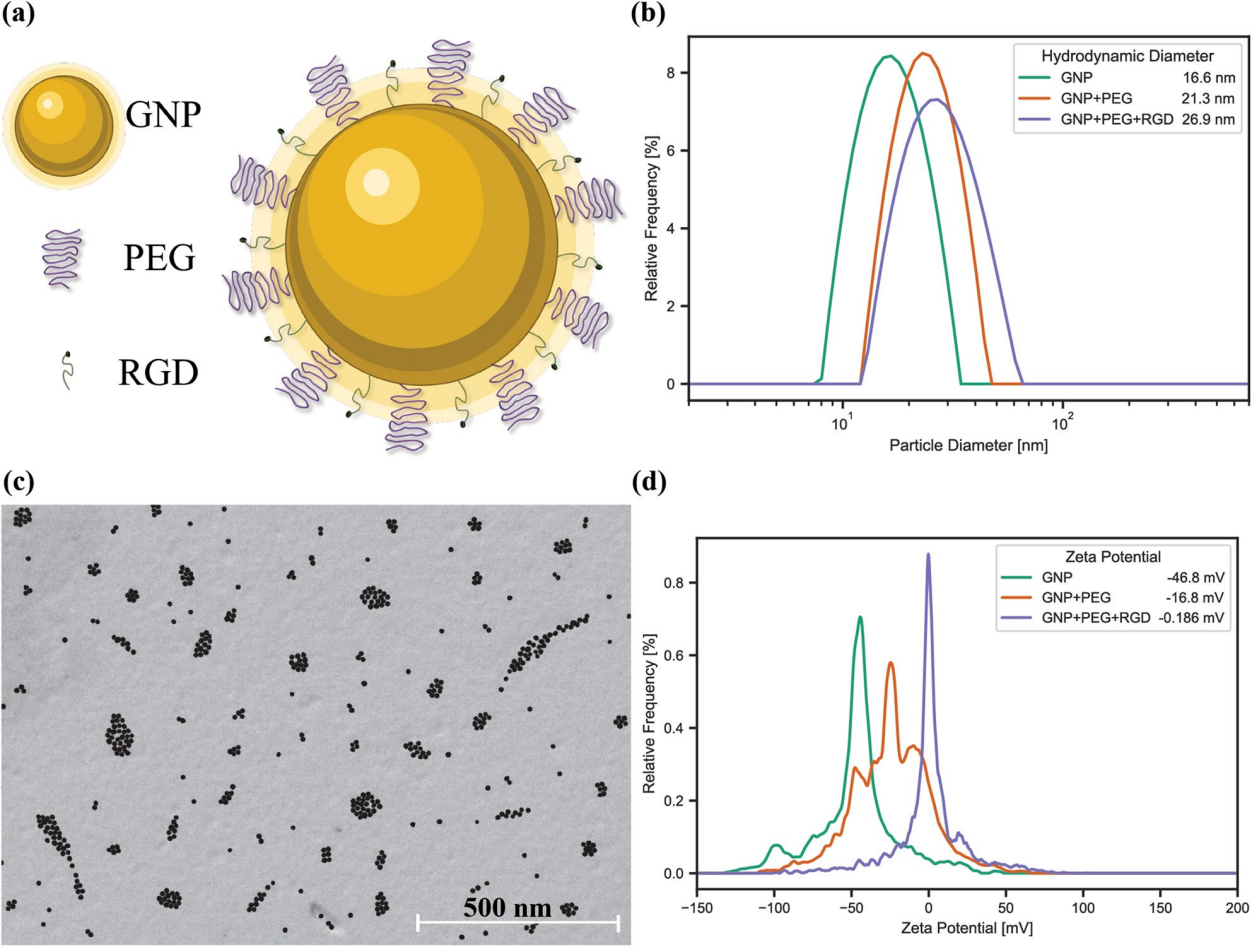
Gold nanoparticle characterization. (**a**) Gold nanoparticles are decorated with polyethylene glycol (PEG) for improved stability and a peptide containing integrin binding domain RGD, to improve uptake into tumours. (**b**) The hydrodynamic diameter of the gold nanoparticles was measured using dynamic light scattering, following functionalization with PEG and RGD. (**c**) Scanning electron microscopy of the gold nanoparticles. Scale bar is 500 nm. (**d**) *ζ*-potential of the gold nanoparticles before and after functionalization with PEG and RGD.

For this study, small spherical GNPs of approximately 13 nm in diameter were conjugated with polyethylene glycol (PEG) and a peptide containing integrin binding domain RGD (Fig. 2a). This functionalized nanoparticle will be referred to as GNP complex. The addition of PEG ensures stability in serum, such as in vivo circulation, while the addition of the RGD peptide improves uptake and can target integrin-overexpressing cells, commonly found in cancer^16,32^. Smaller nanoparticles have been found to more effectively enter cancer cells in more complex spheroid and in vivo environments^27,33^. Measurement of the GNPs before and after conjugation with PEG and RGD was done using dynamic light scattering (DLS; Fig. 2b) which saw the bare GNPs having a hydrodynamic diameter of 16.6 ± 0.33 nm, 21.3 ± 0.43 nm after PEGylation, and 26.9 ± 0.54 nm after addition of RGD peptide, forming GNP complexes. The GNP complexes were also measured using scanning transmission electron microscopy (STEM; Fig. 2c) to verify GNP size, shape, and stability following functionalization. The measured average core size was 12.47 ± 1.21 nm after conjugation with PEG and RGD, while bare GNPs have a measured core size of 12.76 ± 2.8 nm (Supplementary Fig. 1b), signifying no aggregation was present. The difference in the size of the measured diameter in the two modalities is due to the DLS measuring the hydrodynamic diameter, which includes surface ligands, while SEM images only show the dense gold in the nanoparticle core^34^. The size of the GNPs was also measured using UV–Visible spectrometry to estimate the size and concentration, and check stability of the GNP complex (Supplementary Fig. 1c)^35^. The measured size was found to increase from 11.4 ± 0.1 nm to 11.9 ± 0.1 nm following functionalization with PEG and RGD (Supplementary Fig. 1a), further indicating no aggregation and a stable formulation.

The ζ-potential is a useful measure of the suspension stability and can be used to confirm surface adsorption due to different electric potentials of various moieties^36^. To verify conjugation of PEG and RGD, the ζ-potential (Fig. 2d) was measured to be − 46.80 ± 1.24 mV for bare GNPs, − 16.80 ± 0.63 mV for PEGylated GNPs, and − 0.19 ± 0.28 mV for GNP complexes. The increase in the ζ-potential is due to the replacement of the negatively charged citrate molecules on the surface of GNPs with neutral PEG molecules and positively charged RGD peptides. Further, the GNP complex’s stability in serum following conjugation was measured in phosphate buffered saline (PBS; Supplementary Fig. 1d), with a hydrodynamic diameter 28.6 ± 0.55 nm. This indicates that the GNP complexes are stable in highly ionic environments and are suitable for the testing purposes.

### In vitro optimization

To optimize the treatment with GNPs, the 2D monolayer and 3D spheroids were characterized. As seen in Fig. 1, while a 2D model involves single cells, the 3D spheroids introduce a more complex environment, including an extracellular matrix and a drug concentration gradient. For the 3D spheroid, a size of approximately 300–400 μm was chosen as an optimum spheroid size, as larger spheroid sizes (> 350 μm) can introduce a necrotic core and increased number of quiescent cells, affecting results^37^. Different cell lines will have different packing densities and thus will require a varying number of initial cells for spheroid formation; 3125 cells for PC-3 and 780 cells for LNCaP spheroids, as seen in Supplementary Fig. 2. The packing density has been previously shown to affect the efficacy and penetration of anticancer drugs and smaller nanoparticles, and mimicking these in vivo effects is one of the many benefits of using a 3D spheroid model over a 2D monolayer model^38^.

**Figure 3:**
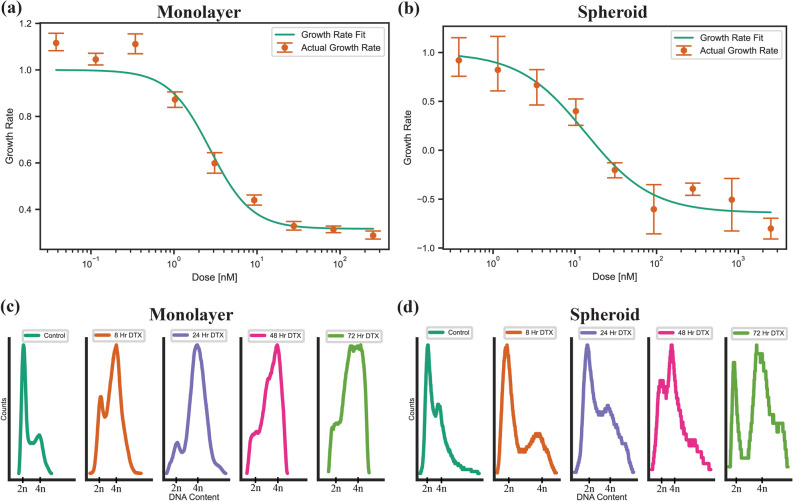
Effects of Docetaxel on two-dimensional and three-dimensional cell models. (**a**,**b**) Proliferation assays for a two-dimensional monolayer and a three-dimensional spheroid, for the prostate cancer cell line PC-3, treated with docetaxel. (**c**,**d**) Cell cycle analysis of a (**c**) monolayer and (**d**) spheroids of PC-3 cells treated with the GR50 dose of docetaxel, calculated per modality.

Docetaxel has been shown to synchronize cells in the G2/M phase of the cell cycle, thus effectively acting as a radiosensitizer, and can also alter the uptake of GNPs in monolayer as well as spheroids^27^. Due to toxicity of DTX at higher doses, the ideal dose will not increase cell death, but rather synchronize the cells effectively to improve uptake of GNPs and act as a radiosensitizer to synergize with the radio sensitizing effects of the gold. To measure this, we used the growth rate inhibition metric to observe the dose in which division time was essentially halted and cytostasis was achieved^39^. For monolayer, this occurred at 2.72 ± 0.21 nM and 0.94 ± 0.034 nM for PC-3 and LNCaP, respectively (Fig. 3a). These doses agree with IC50 values found in literature. For spheroids, the resulting DTX dose showed the higher resistance to treatment inherent to the more complex 3D environment, with PC-3 needing a dose of 13.51 ± 0.31 nM and LNCaP requiring a dose of 3.93 ± 0.50 nM (Fig. 3b). Proliferation data for LNCaP can be seen in Supplementary Fig. 3a, b. Compared to a clinical dose, the area under concentration–time curve (AUC_0→24_) is 65.28 ± 5.04 nM·h and 22.56 ± 0.816 nM·h for a monolayer of PC-3 and LNCaP, respectively, and 324.24 ± 7.44 nM·h and 94.32 ± 12 nM·h for PC-3 and LNCaP spheroids, respectively. A median AUC_0→24_ of 1284 nM·h is observed clinically for a lower dose of 20 mg/m^2^, showing the implemented doses are clinically comparable^40^.

The effects due to the desired doses of DTX were explored using darkfield imaging. DTX causes unregulated formations of microtubules, sequestering them into bundles which hinders the formation of the mitotic spindle necessary for cell division^21^. As a result, in a monolayer of PC-3 cells, compared to control (Supplementary Fig. 4a,b), we can see some multinucleated cells caused by this mechanism of action in the darkfield images, circled in red in Supplementary Fig. 4b. LNCaP monolayer darkfield images can be seen in Supplementary Fig. 5a, b. Spheroids appear to have a slightly disrupted core compared to untreated spheroids (Supplementary Fig. 4c,d.), though the extent is limited, allowing for the bulk of the spheroid to maintain a similar structure. Hyper spectral profiles, from a hyper spectral image, of the cells and background were added to each darkfield image as an inset figure to verify the presence or absence of measured GNPs in the image. Verification of GNP presence through the use of HSI has been widely used in literature, from monolayer to in vivo section analysis^41^. HSI is a useful technique as it allows for each pixel in an image to be associated with a complete reflectance spectral response, with a spectral resolution of 2 nm in the visible-near infrared range. GNPs have a unique reflectance spectral response, due to the surface plasmon resonance of the gold nanoparticles^42^. Thus, using HSI allows us to capture this response and easily identify GNPs compared to tissue or cells. The lines in the image are the mapping of what wavelength corresponds to blue, green, and red for the displayed hyper spectral image. The synchronization effects of DTX in monolayer and in spheroids were then explored using flow cytometry (Fig. 3c,d). DTX has previously been shown to synchronize cell populations in the G2/M phase of the cell cycle, which is the most radiosensitive phase of the cell cycle. For a monolayer of PC-3, there was a fast shift from a normal cell population to a large population of cells in the G2 phase after 8 h, while the shift to a G2-synchronized cell population happened more gradually for spheroids, indicating the chosen dose is working as desired. This effect was maintained for at least 72 h for both cell models. For LNCaP (Supplementary Fig. 3c,d), we did not see a synchronization effect in the monolayer or spheroids, indicating either a resistance in the LNCaP cells to DTX or cells that were affected by DTX failed to transition through S phase to the G2 phase; this has previously been observed in literature^43^.

### Gold nanoparticle uptake in monolayer and spheroid models

**Figure 4:**
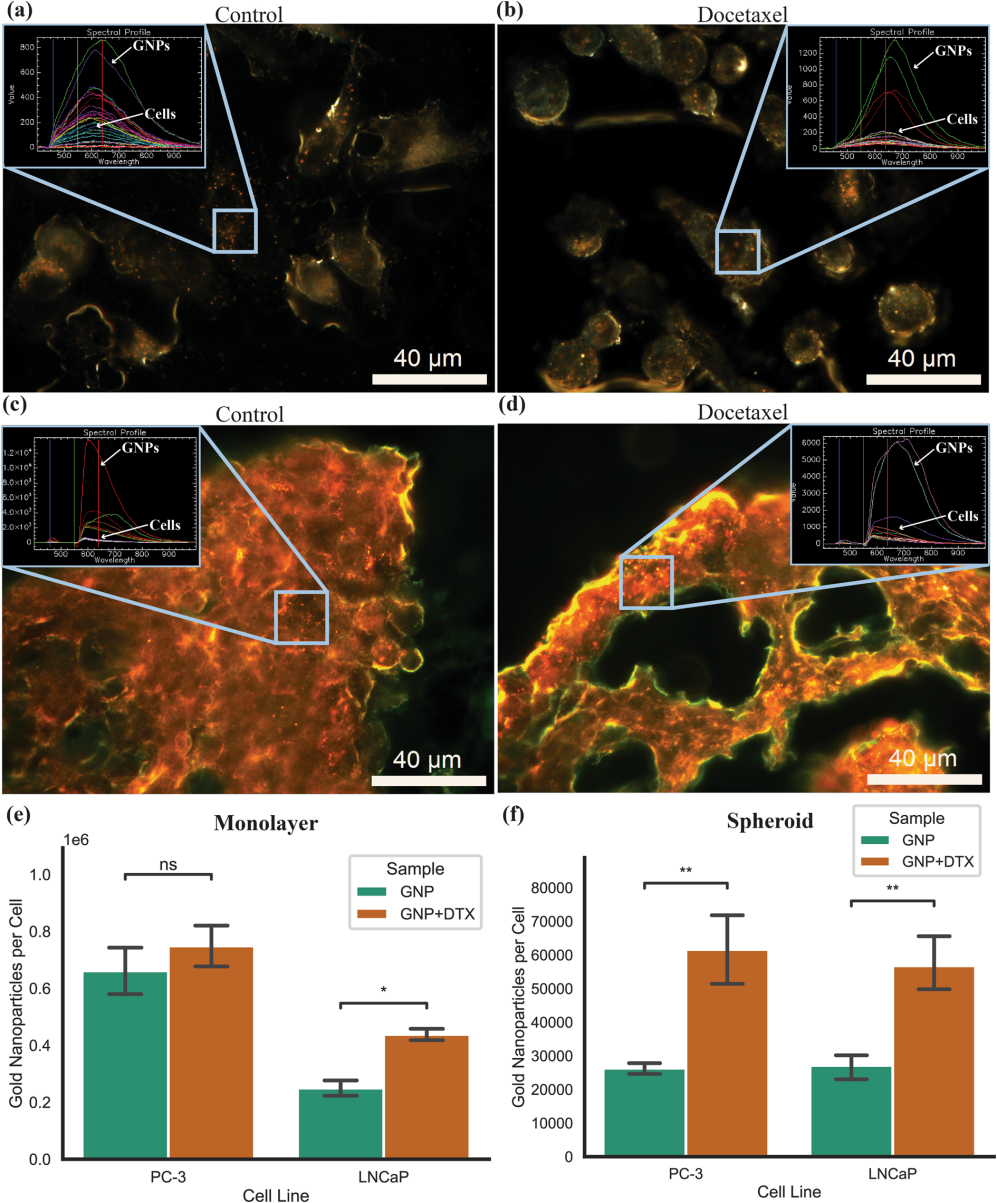
Gold nanoparticle uptake in two-dimensional and three-dimensional models (**a**,**b**) Darkfield images of (**a**) a control and (**b**) a docetaxel-treated monolayer of PC-3 cells. (**c**,**d**) 10 µm spheroid sections of (**c**) control and (**d**) docetaxel-treated PC-3 cells treated with docetaxel and gold nanoparticles. Inset is hyper spectral spectrum of gold nanoparticles and cells. Scale bar is 40 µm. (**e**,**f**) Uptake of gold nanoparticles in (**e**) monolayer and (**f**) spheroids of PC-3 and LNCaP with and without docetaxel. Error bars are the 95% confidence interval from three independent experiments. ns indicates no significance, * indicates 0.01 < *P* < 0.05, ** indicates 0.001 < *P* < 0.01.

To compare the in vitro 2D and 3D models to the in vivo mouse xenograft, we measured the GNP uptake in monolayer as well as in spheroids after treatment with DTX. Both models were treated with GNP complex at a clinically relevant dose of 10 μg/mL for 24 h, which has shown radiosensitization effects before at megavoltage energy ranges in a prostate cancer in vivo model^44^. For the PC-3 cancer cells, after concomitant treatment of DTX and GNP complex, we can see in darkfield images the GNPs accumulate inside the monolayer (Fig. 4a,b) and in the spheroid (Fig. 4c,d) vs control. The inset spectral profile verifies the presence of GNPs in the cells as measured and classified using a spectral angle mapper. It should be noted that the spectral profile will differ from UV–Vis data due to the intracellular agglomeration of the GNPs in endosomes and lysosomes, forcing the GNPs into close proximity and causing an electromagnetic coupling of the plasmon spectra^45^. Darkfield images and hyper spectral profiles for LNCaP can be seen in Supplementary Fig. 6.

We can see in Fig. 4e the measured uptake of the GNP complex in monolayer for both cell lines for control and DTX. There was a 13.2% increase for PC-3 which was not significant, while in LNCaP, there was a significant increase in uptake of 75.4% (p = 0.02392). While LNCaP did not have observed synchronization of the cells, there was still an effect on both cell lines that increased the GNP uptake, although more drastically in LNCaP. In Fig. 4f, the measure uptake of GNP complex in spheroids for both cell lines with and without treatment of DTX is displayed. There was a significant increase in uptake after dosing with DTX of 134% (p = 0.0029) and 109% (p = 0.0010) in both PC-3 and LNCaP spheroids, respectively. Both PC-3 and LNCaP saw beneficial uptake in monolayer and in spheroids with the addition of DTX, with the change in spheroids being more drastic, despite the dose of DTX being normalized to the same growth rate. This may be a result of a higher dose of DTX in spheroids that will disproportionately affect the outer cells, which are the same cells that will have the largest uptake of GNPs as can be seen in the darkfield images. Thus, these cells will experience a larger DTX effect and have an amplified uptake compared to monolayer, where there is a lower dose of DTX and thus less effect. This increased effect is in agreement with previously measured values on separate cell lines^27^. Due to the synchronization of the cells into the G2/M phase with DTX, it is expected for a higher quantity of GNPs to be present relative to other phases, due to having a larger time to accumulate GNPs before division. The process of cell division will lead to the dilution of the per-cell GNP load, which explains the increase in GNPs per cell in samples treated with DTX, which are thus largely in the G2/M phase, compared to control, in which cells are distributed more regularly, in both the monolayer and spheroid samples^46^.

### In vivo effects of Docetaxel

Compared to a monolayer and spheroid, as seen in Fig. 1, the mouse xenograft model introduces a more complex environment including immune infiltrate, circulating blood, and many other cell types in the tumour microenvironment such as fibroblasts and cancer associated fibroblasts. Clinically, doses of DTX of 20 mg/m^2^ have been indicated to have radiosensitizing levels in plasma for up to 1 week and are better tolerated with less side effects compared to higher doses^40,47^. As the goal of this work is to optimize the use of DTX with GNPs in a mouse xenograft for subsequent radiation experimentation, this dosage was also employed in this research. The animal equivalent dose for a mouse xenograft is 6.65 mg/kg; for simplicity, this was rounded to 6 mg/kg^48^. NRG mice were chosen for this study, as they are well suited for cell line derived xenografts and can tolerate higher doses of radiation and chemotherapy treatment. PC-3 was implanted subcutaneously, and treatment to assess biodistribution began once tumours reached a measured volume of 250–300 mm^3^.

**Figure 5:**
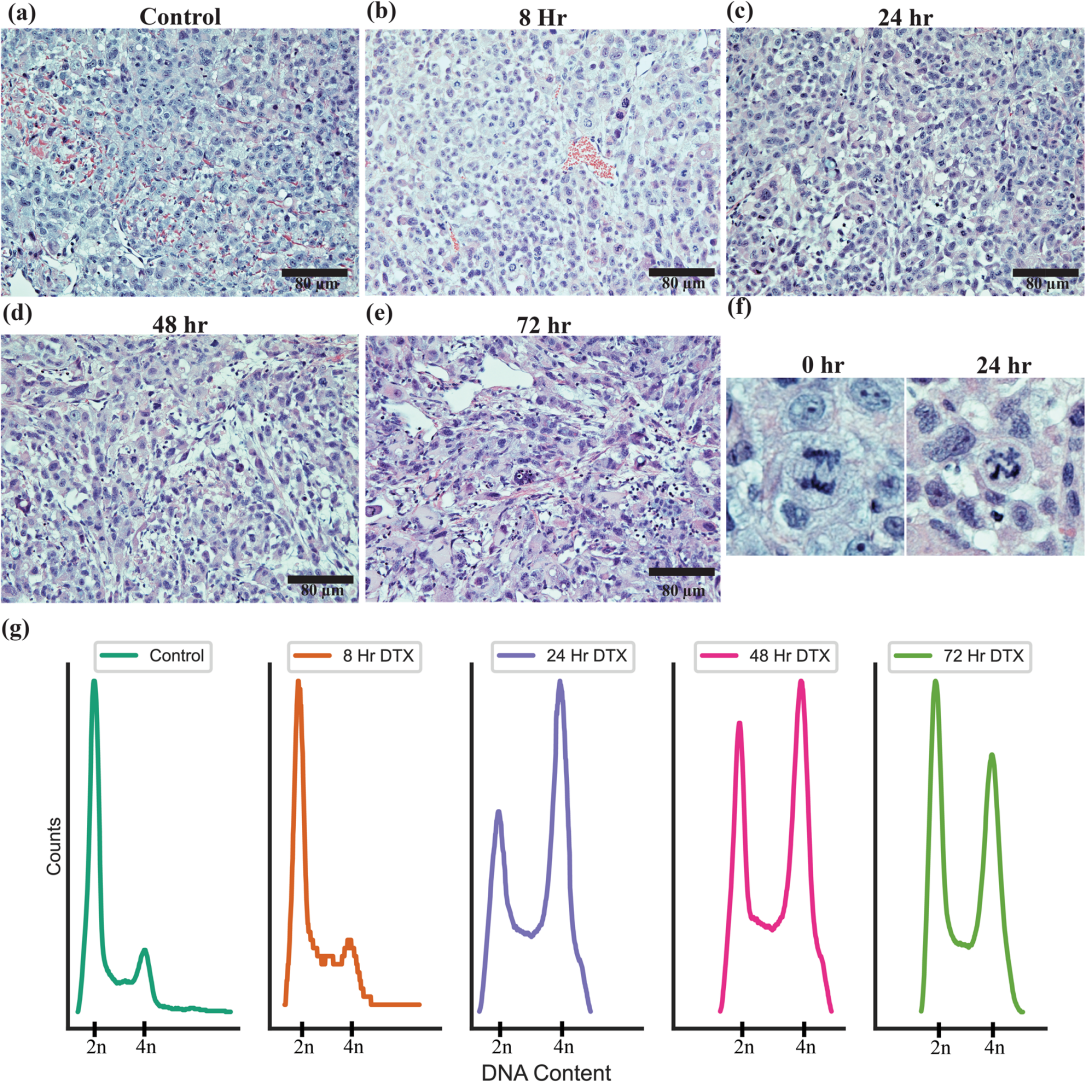
Treatment of PC-3 subcutaneous tumour with docetaxel. (**a**–**e**) Hematoxylin and eosin-stained sections of PC-3 tumour 0, 8, 24, 48, and 72 h after dosing with docetaxel. Scale bar is 80 µm. (**f**) Mitoses in present in the sections of the PC-3 tumour for control and after 24 h of dosing with docetaxel (**g**) Cell cycle analysis of PC-3 tumours after dosing with docetaxel and the subsequent shift in cell cycle synchronization over 0, 8, 24, 48, and 72 h.

To observe the effects of DTX on the tumour microenvironment, tumours were collected after 8, 24, 48, and 72 h of DTX treatment, stained with hematoxylin and eosin (H&E) and imaged as seen in Fig. 5a–e. As time progresses, there appears to be an increase of cellular and nuclear pleomorphism, indicative of altered morphology induced by DTX over time. Further, the mitoses that are occurring appear to be irregular in nature, with increasing condensed chromatin occurring following dosing (Fig. 5f). This is expected due to the nature of DTX and how it acts on cells, as the cells will be synchronized into G2/M and should more regularly exhibit mitotic morphology. As a lower dosage was used, a significant increase in cell death via apoptotic pathways was not observed in the H&E images.

To further verify the increase of G2/M synchronization via DTX dosing, a single cell suspension of the tumour was produced, and the DNA content was measured via flow cytometry. As can be seen in Fig. 5g, after 24 h, most of the cells are in the G2/M phase of the cell cycle in the tumour. After 48 h, this is still the case, though the number of cells in the G1 phase is returning to normal. After 72 h, the cell population is continuing to trend back to G1 phase, with many cells still in G2/M. In the clinic, DTX treatments can be administered weekly in the clinic, and our results suggest the cells will be partially synchronized in the G2/M phase for at least 72 h. This synergizes well with a fractionated radiotherapy treatment regime that has one dose of radiation a day over 5 consecutive weekdays, minimizing the amount of DTX patients can be treated with. Organ H&E images can be seen in Supplementary Fig. 7, and do not present any observable effects with the given dose of DTX.

### In vivo accumulation of gold nanoparticles with Docetaxel

To facilitate the optimum time points for treatment of prostate tumours, the mice were treated intravenously with DTX and GNP complexes with a dose of 1.5 mg/kg. While this is slightly lower than the 10 μg/mL given in vivo (eqv. 10 mg/kg), doses can be scaled in future studies to allow higher radiosensitization effects when radiation is introduced, as GNPs are non-toxic at low doses^49^. Accumulation of GNPs into the tumour is facilitated by a couple mechanisms. First, structural features of solid tumours such as hypervascularization causing a defective vasculature architecture with little supportive tissues, leading to the formation of so-called ‘leaky vessels’, which are pores in the endothelial gaps ranging in diameter from 2 to 100 nm^50^. Combined with impaired lymphatic drainage, the tumours have an enhanced permeability and retention (EPR) effect present which increases the uptake of GNPs and better retains them compared to normal, healthy tissue. Further, due to the functionalization of the GNPs with RGD, we expect even further targeting of tumours, due to the increased expression of integrin dimers—i.e. αvβ3, αvβ5, and α5β1, which can recognize the RGD motif – in various cancers including PC-3 and LNCaP ^51^.

**Figure 6:**
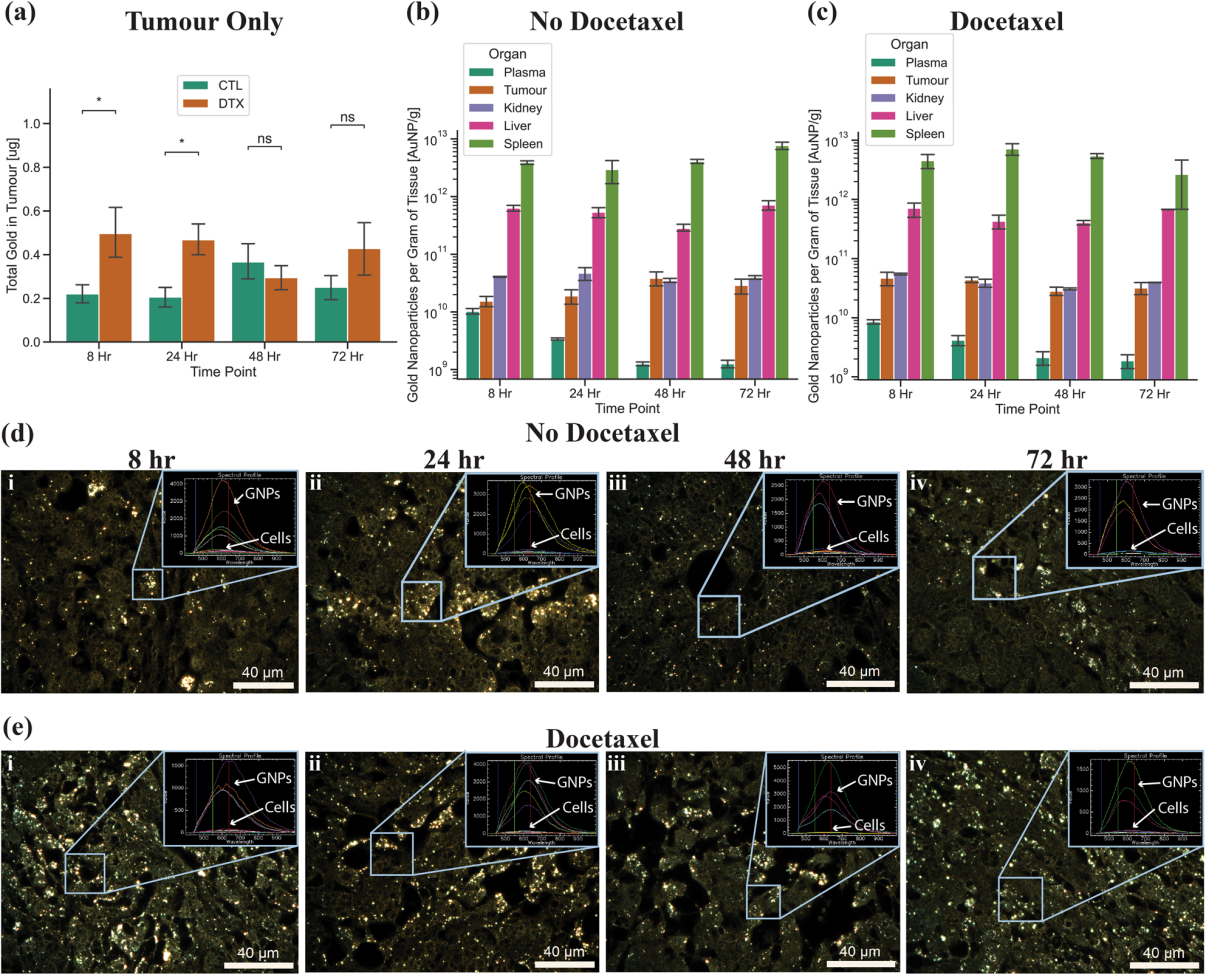
Gold nanoparticle uptake in in vivo mouse xenograft model. (**a**) Total gold uptake in the tumour after 8, 24, 48, and 72 h for tumours treated with gold or treated with gold and docetaxel. DTX refers to docetaxel treated samples and CTL refers to control samples with no docetaxel. (**b**,**c**) Total gold nanoparticles per gram of tissue for plasma, tumour, kidney, liver, and spleen after treatment (**b**) without docetaxel and (**c**) with docetaxel, after 8, 24, 38, and 72 h. Error bars are the 95% confidence interval from four independent mice. ns indicates no significance, * indicates 0.01 < p < 0.05. (**d**,**e**) Darkfield images of 5 µm sections of tumour tissue 8, 24, 48, and 72 h after (**d**) gold nanoparticle treatment and after (**e**) concurrent gold nanoparticle and docetaxel treatment. Inset is hyper spectral spectrum of gold nanoparticles and cells. Scale bar is 40 µm.

Comparing the tumour when treated with GNP complex alone to GNP complex and DTX (Fig. 6a), we found that there was a significant increase in uptake of GNP complex after 8 h and 24 h of dosing with DTX of 125% (p = 0.045) and 126% (p = 0.013). However, there was no significant increase after 48 and 72 h, which corresponds with the reduction of cells synchronized in the G2/M phase of the cell cycle. The increase in uptake of GNPs is due to the same mechanism that caused an increase in monolayer and spheroids, mainly the synchronization of cells into the G2/M phase. This facilitates a higher per-cell uptake compared to other phases in the cell cycle, allowing for more GNPs in each cell to be utilized in future radiotherapy studies. When looking at the control organs (Fig. 6b) and organs treated with DTX (Fig. 6c), when normalized to GNPs per gram of tissue, we can see that there is a relative increase in GNP uptake in the tumour compared to the rest of the organs. For control, we found that after 8, 24, 48, and 72 h, 0.52%, 0.46%, 0.79%, and 0.58% of the deposited dose is accumulated within the tumour, respectively, while with DTX, after 8, 24, 48, and 72 h, 1.13%, 1.02%, 0.68%, and 0.70% respectively accumulated in the tumour. Further, the DTX does not significantly increase the deposited dose of GNPs into the normal organs. This indicates that there is a short-term increase in the GNP uptake due to the DTX that maintains a higher total accumulation in the tumour relative to control over 72 h. When moving to radiation, this is an ideal result, indicating that both the DTX and the GNPs will stay inside the tumour, allowing for a synergistic radiosensitization effect up to 72 h after initial dosing, and indicating the best time for the first dose of radiation is 24 h after initial treatment.

To visualize the GNP uptake into the tumour, darkfield images were taken for all time points. For control tissue samples (Fig. 6d), there does not appear to be large difference between all four time points, although the amount of gold appears higher in the 8-h and 24-h time point. However, when compared to samples treated with DTX (Fig. 6e), we can immediately see a visual increase in the amount of gold. This agrees with the quantified increase in uptake of GNPs found previously. Darkfield images of organs without and with DTX can be seen in Supplementary Fig. 8 and Supplementary Fig. 9. A result that was observed consistently in the tumour sections was a large quantity of GNPs around the blood vessels. When comparing the darkfield images of the tumours to that of the spheroids (where the media acts as a blood vessel), we see a similar effect, where there is a concentration gradient acting on the GNPs, reducing the penetration depth. Further, compared to the PC-3 spheroid model, which had a 130% increase in GNP uptake, there was a very similar 126% increase in the tumour after 24 h. These results highlight the benefits of using a spheroid model over a monolayer model to facilitate a quick translation of GNPs to the clinic.

## Conclusion

Improving activity against cancerous tumour tissue while preserving normal tissue and reducing toxicity is achievable with the use of radiosensitizers like GNPs. To further synergize this effect, the addition of docetaxel can allow for an improved therapeutic effect within the tumour^52^. By escalating the dose of radiation within a tumour while minimizing the dose to normal tissue, with targeting of tumours with radiosensitizers, we can improve patient outcomes and quality of life. Before radiation can be introduced, however, the combination of GNPs and DTX must be optimized. Thus, in this work, we measured the alteration in uptake of GNPs following treatment with DTX in vitro, in the 2D monolayer model and the 3D spheroid model, followed by an in vivo mouse xenograft, using prostate cancer cell lines PC3 and LNCaP. In spheroids and the mouse xenograft, it was found that with the addition of DTX, there was a significant increase in the gold uptake relative to no DTX, and that the two different cell models mimicked each other in both the relative increase of gold as well as the distribution of gold, while the monolayer of cells did not show similar effects. These results are the building blocks for the use of radiation with DTX and GNPs in an in vivo mouse xenograft and highlight the benefits of using an in vitro model such as the 3D spheroids that can better mimic the tumor microenvironment. By optimizing at a 3D level and comparing to an in vivo model, we can enable swifter RT experiments with higher prospects of future treatment success.

## Materials and methods

### Cells and culture conditions

PC-3 and LNCaP, both human prostate cancer cell lines, were purchased from the American Type Culture Centre (ATCC) and have catalogue numbers CRL-1435 and CRL-1740 respectively. PC3 was cultured in Dulbecco’s Modified Eagle Medium (DMEM; Gibco) supplemented with 10% Fetal Bovine Serum (Gibco), 1% Penicillin and Streptomycin (Gibco), and 4 mM GlutaMax (Gibco). LNCaP was cultured with RPMI-1640 Medium (Gibco), supplemented with 10% Fetal Bovine Serum (Gibco), 1% Penicillin and Streptomycin (Gibco), and 2 mM GlutaMax (Gibco). For cell dissociation, TrpyLE (Gibco) was used.

### Synthesis, surface modification, and characterization of gold nanoparticles

Gold NPs of size ~ 17 nm were synthesized using the citrate reduction method^53^. This was accomplished by adding 1.18 mL of 1% HAuCl_4_⋅3H_2_ O (Sigma-Aldrich) to 28.82 mL of double–distilled water and heated on a hot plate while stirring vigorously. Once it reached the boiling point 1.2 mL of 5% sodium citrate tribasic dihydrate (HOC(COONa) (CH_2_ COONa)_2_·2H_2_O; Sigma-Aldrich) was added and mixed. Once the color of the solution changed from dark blue to red, the solution was left to boil for another ten minutes while stirring. Finally, the GNP solution was brought to room temperature while stirring.

The GNPs were PEGylated using PEG of size 2000 Da, along with an RGD peptide of size 1600 Da. PEG was stirred into the GNP solutions such that the grafting density will be 1 PEG molecule per 1 nm^2^ of surface area. For 17 nm GNPs, this results in 907 PEG per GNP to be added to the solution, respectively. Following PEGylation of GNPs, the peptide containing integrin binding domain RGD (CKKKKKKGGRGDMFG) was added to PEGylated GNPs at a ratio of 1 RGD molecule per 2 PEG molecules, referred through this paper as GNP complex.

GNPs were characterized using ultraviolet–visible (UV–VIS) spectrometry (Perkin Elmer λ Spectrophotometer) for approximate size and concentration estimates. Further, dynamic light scattering (DLS), and ζ-potential (Anton Paar LiteSizer 500) were utilized to measure the hydrodynamic diameter and the surface charge of the particles. Stability of the GNPs was measured in phosphate buffered saline (PBS). Imaging of the GNPs with scanning transmission electron microscopy (STEM; Hitachi SU9000) was used to verify the diameter of the GNPs. Brightfield, secondary electron, and high-angle annular dark-field images of each location were taken using 14 keV STEM. Brightfield images were used for GNP diameter measurement purposes.

### Cell culture and growth of spheroids

For 2D and 3D cell models, all cells were initially split from a monolayer of cells at approximately 80% confluence. For the 2D monolayer cell model, cells were plated such that final confluence at the end point is approximately 70% in either six-well plates or 96-well plates, with initial cell count dependent on the experiment. Once plated, cells are left in the incubator at 37 °C and 5% CO_2_ for 24 h to ensure adherence, after which experiments are initiated.

For 3D spheroid cell models, cells are plated in ultra-low attachment 96-well microplates (Corning), with 1500 cells for PC-3 per well and 1000 LNCaP cells per well. For PC-3, the media was supplemented with 3% Geltrex matrix (Gibco) on ice, ensuring the temperature did not rise above 15 °C. The cells are then centrifuged at 350xg for 5 min, at 4 °C and left in the incubator at 37 °C and 5% CO_2_. Experiments are initiated once the spheroids form, after incubation for approximately 72 h.

### Proliferation assay of Docetaxel in monolayer and spheroids

For the 2D monolayer cell model, 10,000 cells from each cell line were plated in 96 well black clear bottom microplates (Greiner), leaving one column empty for control. Once adhered, each column (8 wells) was treated with a unique Docetaxel dose ranging from 250 nM to 1.27 × 10^–2^ nM in media, ensuring that the concentration of dimethyl sulfoxide (DMSO) in which the Docetaxel (DTX) is stored, is constant in all columns. Similarly, for the 3D spheroid cell model, once formed, each column (8 wells) was treated with a unique DTX dose ranging from 2500 nM down to 0.254 nM. This was completed in triplicate, on three different plates per cell line. At the same time, a control column without treatment of DMSO or DTX was measured using PrestoBlue Cell Viability Reagent (Invitrogen). A 10% solution of PrestoBlue in DMEM or RPMI, depending on the cell line, was added to the cells and left in the incubator for 20 min. The resulting fluorescence was measured using a Cytation 1 Cell Imaging Multi-Mode Reader (BioTek) for the single column.

For monolayers, the media was removed from each sample after 24 h, rinsed twice with phosphate buffered saline (PBS; Invitrogen), and left to incubate for a further 48 h in fresh media. For spheroids, half of the media was removed carefully after 24 h, and then rinsed 5 times with PBS, ensuring approximately a 162 × dilution from the initial concentration. Finally, the media was replaced and left for a further 48 h in the incubator.

After 48 h, all the media for the 2D monolayer model was removed and treated with 10% PrestoBlue in media assay, following the manufacturers protocols. Plates were left in the incubator, and fluorescence was measured after 20 min using the Cytation 1. Similarly, for the 3D spheroid model, the media was removed until 100 µL remained, ensuring that the sample was not aspirated, and then 100 µL of 20% PrestoBlue in media was added to make a final concentration of 10% PrestoBlue. The plates were then left in the incubator and fluorescence was measured after 4 h using the Cytation 1.

### Animal xenograft model

Male NRC mice (ages six to ten weeks old) were purchased from the BC Cancer Research Institute for the in vivo experiments (GNP uptake with and without DTX). The methodology has been used, in general, falls within a service-oriented Animal Care Protocol that has been reviewed and approved by the Institutional Animal Care Committee (IACC) at the University of British Columbia. During the study the care, housing and use of animals will be performed in accordance with the Canadian Council on Animal Care Guidelines. Reporting of animal data in this study followed the recommendations set out in the ARRIVE guidelines.

### Cell preparation and implantation into mice

Cells are started from a frozen vial of lab stock which were frozen down from the ATCC original vial, tested for mycoplasma negative and kept in lab liquid nitrogen tanks. Cell cultures with passage #3 to #10 and a confluence of 80–90% were harvested for in vivo studies. Cells were grown in Ham’s F12 (Gibco) medium supplemented with 2 mM L-glutamine (Gibco) and 10% FBS (Gibco) at 37 °C in 5% CO_2_ environment. Cells are sub-cultured once a week with split ratio 1:3 to 1:6 and expanded. The medium needed to be renewed once a week.

Cells were rinsed briefly one time with 2 ml of fresh Trypsin/EDTA solution (0.25% trypsin with EDTA 4Na) and aspirated. Then 1.5 ml of Trypsin/EDTA was added and the cells were allowed to dissociate with the flask at 37 °C for a few minutes. Fresh medium was added to the cells, and 50 μl of cell suspension was mixed with Trypan Blue (1:1) and the cell count and cell viability on a cellometer Auto4 was measured. The cells were then centrifuged at 200 × g for 7 min and the supernatant aspirated. Re-suspend the cells in cold growth medium to 2 times of desired concentration (50 × 10^6^ /ml), and the mix on ice with Matrigel (1:1). The cell number for inoculation is 5 × 10^6^ cells in an injection volume of 100 μl per animal. All equipment coming into contact with Matrigel (needles, syringes, pipette tips) were chilled prior to injection.

On study day 0, 5 × 10^6^ tumour cells in a volume of 100 μL were implanted subcutaneously into each animal’s flank using a 28-gauge needle. Tumour growth was monitored by measuring tumour dimensions with calipers beginning on first day of treatment. Tumour length and width measurements were obtained every Monday, Wednesday and Friday. Tumour volumes were calculated according to the equation L X W^2^ X 0.5 with the length (mm) being the longer axis of the tumour. Animals were also be weighed at the time of tumour measurement. Tumours were allowed to grow to a maximum of 800 mm^3^ before euthanasia All animals were observed daily for general health. In particular, signs of ill health were based on body weight loss, change in appetite, behavioural changes such as altered gait, lethargy and gross manifestations of stress. If signs tumour-related illness were seen, the animals would have been euthanized (isoflurane anesthesia then CO_2_ asphyxiation).

### Gold nanoparticle and docetaxel treatment of mouse PC-3 xenograft model

Treatment of mice began once the tumours were measured to be about 250–300 mm^3^. For this study, Docetaxel (Sandoz) was provided in 10 mg/mL solution, containing 96% Citric Acid, ethanol, PEG 300, and polysorbate 80. Unopened vials were kept at 2–25 °C and protected from light. The GNP complex solution was prepared as described and stored in 4 °C while protected from light. The dose for DTX was 6 mg/kg of mouse, administered intravenously, while the dose for GNP complex was 1.5 mg/kg of mouse, and was also administered intravenously.

The mice were broken up into 3 study groups: a) untreated, b) GNP complex only, and c) DTX + GNP complex, administered concurrently. For group a), 6 mice were allocated to measure PC-3 tumour growth kinetics. For groups b) and c), 16 mice were allocated for each group, with 4 mice per four different time points: 8, 24, 48, and 72 h after dosing.

### Pharmacokinetic tissue sampling

After mice are injected with the test articles (GNPs or DTX + GNP complex) and the appropriate amount of time has passed, cardiac puncture and tissue harvest were performed. For each time point, 3 out of 4 mice were used for histopathology, where 50% of the tumour was placed in 10% neutral buffered formalin, and the other 50% will be frozen for use in the biodistribution study. Further, 3 out of 4 of the mice had the spleen, both kidneys, the liver, and blood plasma frozen for use in the biodistribution study. The entire tumour of each remaining mouse from each time point was placed in 70% ethanol for further cell cycle analysis, kept at − 20 °C, and their blood taken for Complete Blood Count with Differential and blood chemistry (performed at IDEXX), while their organs were also placed in 10% neutral buffered formalin for histopathology.

### Cellular uptake of gold nanoparticle complexes

Incubation with GNP complex was done at a concentration of 10 μg/mL for all the stated experiments in monolayer and in spheroids. To test the effect that DTX had on the uptake of the GNPs, the different cell models and cell lines were dosed with the desired concentration found in the cell proliferation experiments concurrently with GNP dosing. This correlated to a dose of 0.944 nM for a monolayer of LNCaP, a 3.933 nM for a spheroid of LNCaP, a dose of 2.722 nM for a monolayer of PC-3 and a dose of 13.51 nM for a spheroid of PC-3. Samples were incubated at 37 °C and 5% CO_2_ for 24 h following treatment. Cells were then rinsed with PBS at least three times, ensuring no sample is lost aspirated, and dissociated with TrypLE. For spheroid samples, the cells were left in TrypLE for 30 min at 37 °C to improve dissociation and produce a single-cell homogenous solution, while the monolayer samples were left in TrypLE for approximately 5 min. The cells were counted using a Coulter Counter (Z2 Coulter; Beckman Coulter) for GNP quantification per cell.

For the harvested in vivo samples, the tissue had to be disassociated first. To accomplish this, each sample was weighed and then placed in 2 mL of TrypLE and blended using a handheld homogenizer (Fisher). These samples were then diluted to 5 mL in Millipore water and treated with aqua regia along with the monolayer and spheroids, as follows.

To measure the gold content for each sample, 500 μL of each sample were treated with 250 μL aqua regia (3:1 ratio of HCl:HNO_3_(VWR)) in a 90 °C mineral oil bath for a minimum of 30 min. After, 100 μL of hydrogen peroxide (VWR) was added and the samples were returned to the oil bath for 30 min, ensuring complete cell breakdown. These samples were then diluted down to 2.5% v/v acid content in deionized water and the gold content was quantified using inductively coupled plasma mass spectrometry (ICP-MS; Agilent 8800 Triple Quadrupole). In vivo samples from the mice were filtered first in a 0.2-micron filter (Fisher). Calculation of nanoparticle content based on absolute gold content can be seen in previous publications ^54^. Gold content for mice samples was calculated based off mass instead of cell count.

### Histopathology

10% neutral buffered formalin-fixed tissues were placed in labeled tissue processing cassettes and processed overnight into paraffin using an automated tissue processor (Leica ASP6025S). Processed tissues were then embedded into paraffin blocks and sectioned at a thickness of 4 μm. Sections from three levels spaced 50 μm apart were collected and mounted onto microscope slides (two slides per level; one for Hematoxylin & Eosin staining and another for coverslipping only).

After air drying overnight, the slides were loaded into a robotic stainer and coverslipper (Autostainer XL and CV5030 coverslipper; Leica), baked at 65 °C for 15 min and then de-waxed in xylene. Half of the slides were then coverslipped with a resinous mounting medium for darkfield imaging and the other half were stained with Hematoxylin & Eosin (H&E) according to standard procedures for brightfield images.

### Preparation of cells for imaging using darkfield and hyper spectral imaging

To prepare cells for darkfield imaging for 2D monolayer samples, all cell lines were plated in a six-well plate with glass coverslips placed on the bottom of each well. The cells were then treated as described previously using GNP complex and docetaxel at the desired doses. Following treatment, the cells were rinsed three times with PBS and fixed using 4% paraformaldehyde (PFA) for 20 min at 37 °C. The cover slips were then removed from each well and mounted to a glass slide using Permount Mounting Medium (Fisher).

Similarly, for 3D spheroid samples, following treatment of spheroids with GNP complex and docetaxel, the cells were cleaned 5 × to dilute GNPs down dramatically, fixed in paraformaldehyde for 30 min at 37 °C, and then embedded in optical cutting temperature (OCT) compound (Fisher) and left in a − 20 °C freezer. The spheroids were then sectioned at 10 μm using a cryostat (Leica) and adhered to a charged microscope slide. The sections are washed with acetone to remove excess OCT, then rinsed with water. The slides are then stained with Eosin (SelecTech; Leica) stain for 30 s and washed three times with 100% ethanol. Finally, the cells were rinsed three times with xylenes, coverslipped, were mounted onto the glass slides using Permount, and dried overnight for microscopy. Each sample, either monolayer or spheroid, was imaged using darkfield microscopy and hyper spectral imaging (HSI; CytoViva) under a 10X and 60X objective. The depth of the GNPs from the surface of each spheroid section sample was measured using ImageJ. A minimum of 100 measurements of depth were taken for each sample using the spheroids imaged with the 10 × objective.

### Cell cycle analysis of Docetaxel using flow cytometry

Docetaxel was administered to the monolayer and spheroid cells at desired concentrations as found in proliferation assay. After set time points of 0, 1, 4, 8, and 24 h, the cells were harvested as described previously using TrypLE, and a single cell suspension was formed. Cells were washed with PBS and centrifuged at 300xg for 5 min twice. The cell pellet was then re-suspended in 1% PFA in PBS for fixation and incubated on ice for 15 min. Cells were again washed in PBS and centrifuged at 350xg for 5 min. Cells were re-suspended in 0.3 mL PBS and 0.7 mL freezer cold 100% ethanol (overall 70% ethanol). Samples were incubated in the dark at 4 °C for at least an hour, further fixing and dehydrating the cell sample. For tumour samples, the tumours were treated in Collagenase/Dispase (Roche) according to manufacturer’s instructions for two hours. Following this, the solutions were filtered through a 100-micron cell strainer and treated with the monolayer and spheroid samples.

Samples were then centrifuged at 350xg for 10 min at 20 °C. The cell pellet was re-suspended in 1 mL of 0.5% bovine serum albumin (BSA) in PBS, denoted as PBS/BSA and centrifuged at 350xg for 5 min at 20 °C. To permeabilize the cell membrane and degrade RNA, the cell pellet was re-suspended in PBTB (PBS, 0.5% BSA, 0.1% Triton-X 100) followed by an addition of RNaseA at a concentration of 100 ug/mL. Samples were then left to shake at 37 °C for 25 min. For labelling DNA, tubes were covered in foil, propidium iodide (PI) added at a concentration of 10 μg/mL and incubated on a shaker at 4 °C for at least 1 h. The cells were then centrifuged at 350xg for 5 min at 20 °C. Finally, we re-suspended PI-stained cells in 1 ml of PBS/BSA and passed the solution through a 50 μm cell strainer before running on Flow Cytometer (BD FACS Calibur). Propidium iodide is highly fluorescent at 488 nm with broad emission centered around 600 nm. The amount of DNA content indicates which phase the cell population is in, and thus how synchronized it is.

### Statistical analysis

A Welch's independent t-test with Bonferroni correction was preformed using the statannot python package. A p value < 0.05 was considered statistically significant. Experiments were repeated three times and the data presented is the average, for all experiments.

### Ethics approval

The methodology described here has been described, in general, within a service oriented Animal Care Protocol that has been reviewed and approved by the Institutional Animal Care Committee (IACC) at UBC—protocol #A18-0276. This study protocol will be made available to the IACC on requested and under a confidentiality agreement. All data collected and reported on this study protocol will be made available to the IACC on requested and under a confidentiality agreement. During the study the care, housing and use of animals will be performed in accordance with the Canadian Council on Animal Care Guidelines.

### Ethics guidelines

Reporting of animal data in this study followed the recommendations set out in the ARRIVE guidelines.

## Supplementary Information

Supplementary Information.

## Data Availability

The datasets used and/or analysed during the current study available from the corresponding author on reasonable request.
