# Supplementary material for: Enhancing nanoparticle accumulation in two dimensional, three dimensional, and xenograft mouse cancer cell models in the presence of docetaxel
Source: Sci Rep. 2022 Aug 5;12:13508. doi: 10.1038/s41598-022-17752-5 (PMC9356051; doi:10.1038/s41598-022-17752-5)
Supplement: Supplementary file 1 — Supplementary Information. [file 41598_2022_17752_MOESM1_ESM.pdf]

Enhancing nanoparticle accumulation in two dimensional, three dimensional, and xenograft mouse cancer cell models in the presence of docetaxel

Kyle Bromma[1], Nancy Dos Santos[2], Ingrid Barta[3], Abraham Alexander[1,4], Wayne Beckham[1,5], Sunil Krishnan[6] and Devika B. Chithrani[1,5,7,8]*

1 Department of Physics and Astronomy, University of Victoria, Victoria, BC, Canada.
2 British Columbia Cancer Research Institute, Vancouver, BC, Canada.
3 Animal Care Services, University of British Columbia, Vancouver, BC, Canada.
4 Department of Surgery, University of British Columbia, Vancouver, BC, Canada.
5 British Columbia Cancer, Victoria, BC, Canada.
6 Mayo Clinic, Florida, United States.
7 Centre for Advanced Materials and Related Technologies (CAMTEC), University of Victoria, Victoria, BC, Canada.
8 Centre for Biomedical Research, University of Victoria, Victoria, BC, Canada.

* Corresponding authoer. E-mail: devikac@uvic.ca

<u>**Supplementary Figure 1**</u>

(a)

Sample	Calculated UV-Vis Size [nm]	Hydrodynamic Diameter [nm]	Zeta Potential [mV]
15 nm	11.39 ± 0.056	16.6 ± 0.33	-46.80 ± 1.24
15 nm + PEG	11.85 ± 0.059	21.3 ± 0.43	-16.80 ± 0.63
15 nm + PEG + RGD	11.86 ± 0.059	26.9 ± 0.54	-0.19 ± 0.28

(b) **(c)** **(d)**

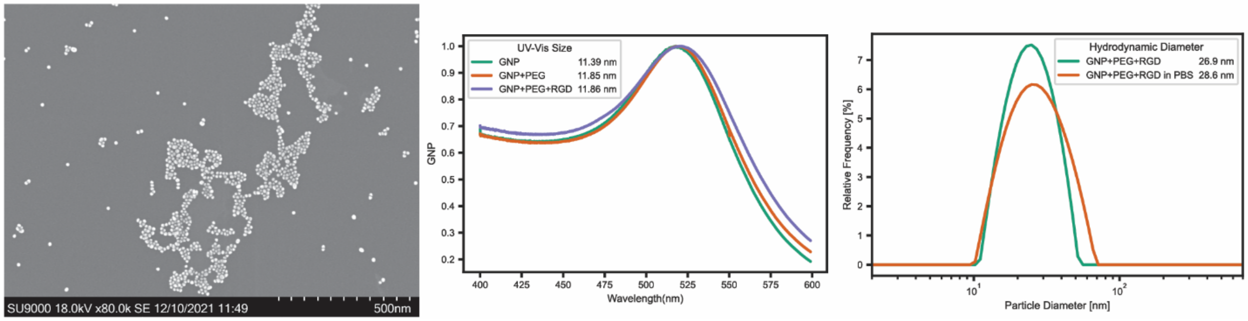

Figure 1. *Gold nanoparticle characterization.* (a) Table of calculated sizes calculated with UV-Visible spectrometry, the hydrodynamic diameter calculated by dynamic light scattering, and the zeta potential of the different formulations of gold nanoparticles. (b) Gold nanoparticles imaged using a scanning electron microscope without PEG and RGD.d (c) UV-Visible spectrum of bare gold nanoparticles, gold nanoparticles decorated with PEG, and gold nanoparticles decorated with PEG and RGD. (d) Gold nanoparticles with PEG and RGD in water and in PBS.

Supplementary Figure 2

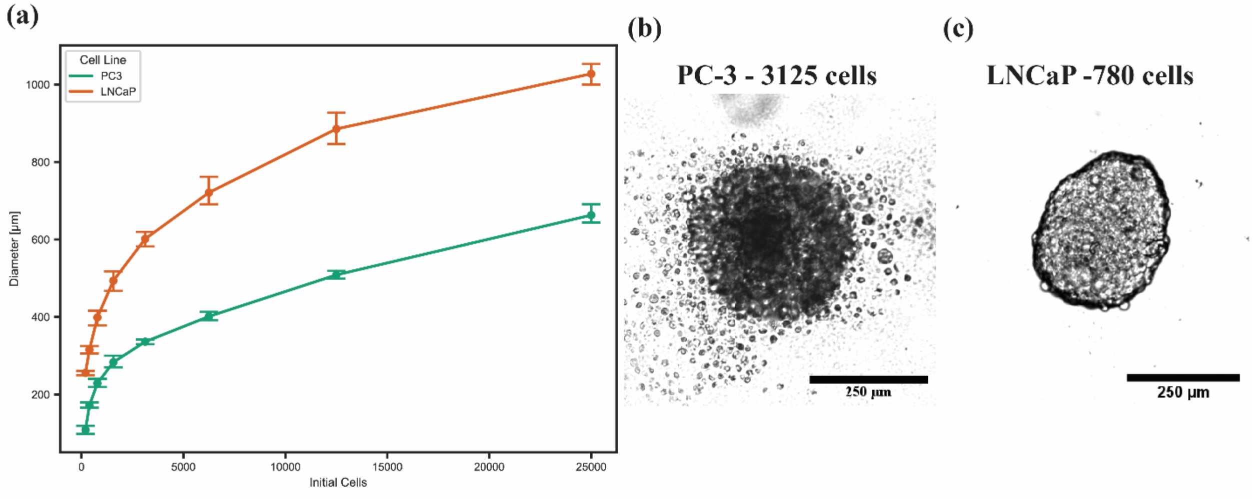

Figure 2. *Characterizing spheroid size.* (a) Size of the spheroids for the two prostate cancer cell lines under different initial cell count conditions. An approximate size of 300-400 µm was used for all experiments. (b,c) Brightfield images of the spheroids under different initial cell counts, scale bar is 250 µm.

Supplementary Figure 3

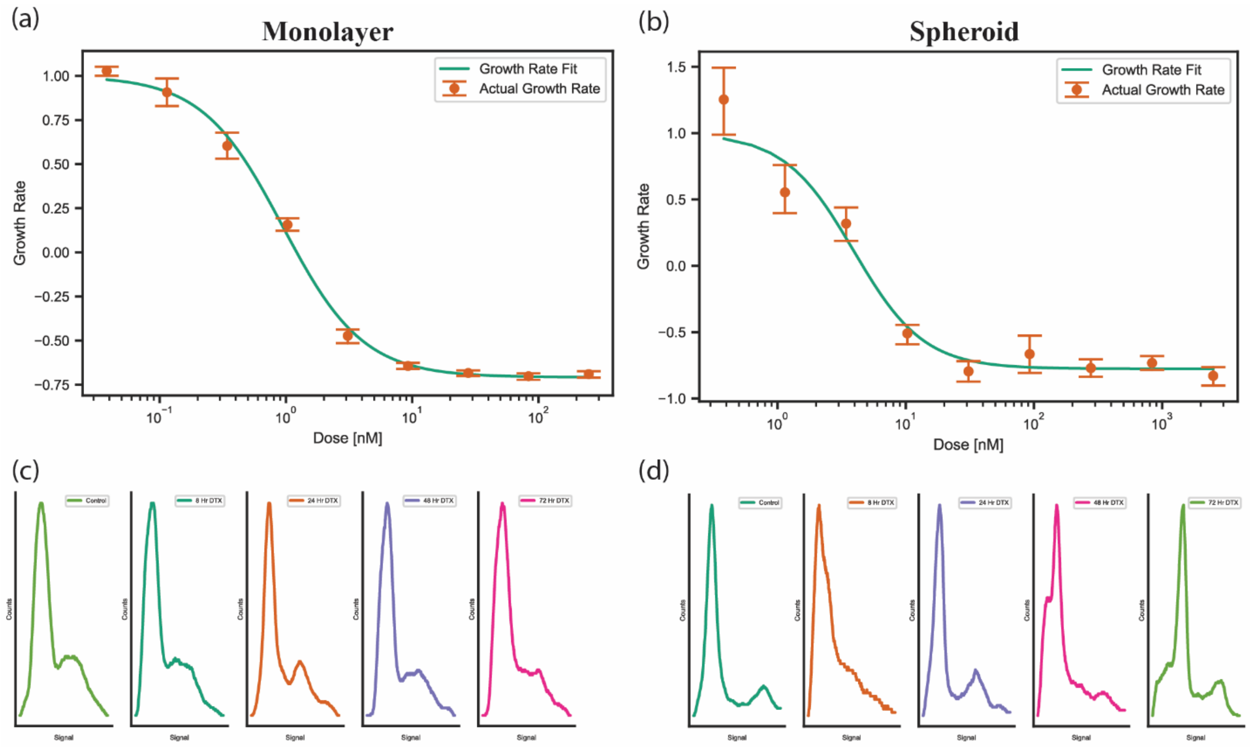

Figure 3. *Effects of Docetaxel on two-dimensional and three-dimensional cell models.* (a,b) Proliferation assays for a two-dimensional monolayer and a three-dimensional spheroid, for the prostate cancer cell line LNCaP, treated with docetaxel. (c,d) Cell cycle analysis of a (c) monolayer and (d) spheroids of LNCaP cells treated with the GR50 dose of docetaxel, calculated per modality.

Supplementary Figure 4

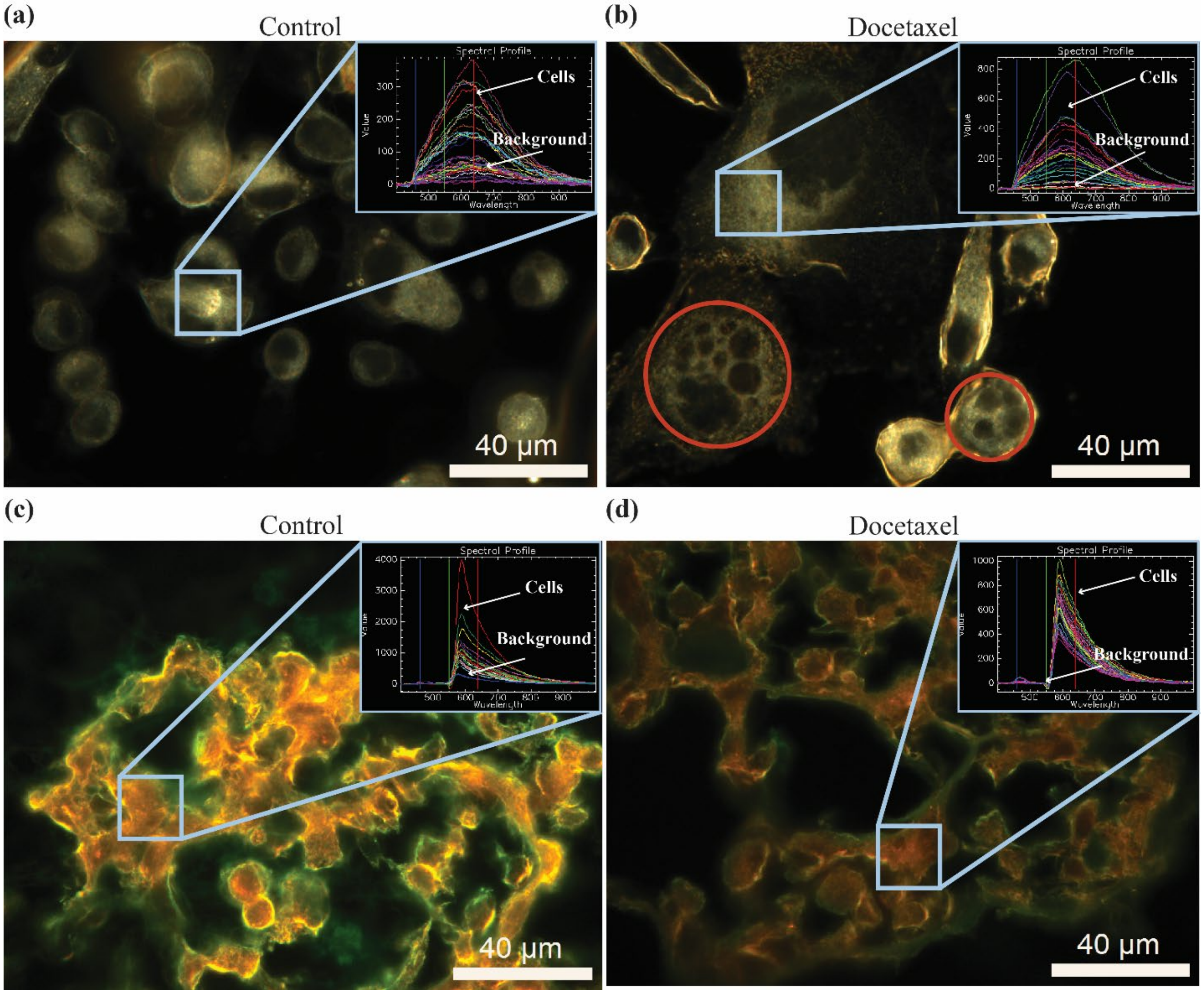

Figure 4. *Darkfield images of monolayer and spheroids.* (a,b) Darkfield images of a monolayer of PC-3 (a) without and (b) with docetaxel. Cells with multinucleated cells due to docetaxel have been circled in red on (b). (c,d) Darkfield images of 10 µm sections of PC-3 spheroids (c) without and (d) with docetaxel. Inset is hyper spectral spectrum of cells. Scale bar is 40 µm.

Supplementary Figure 5

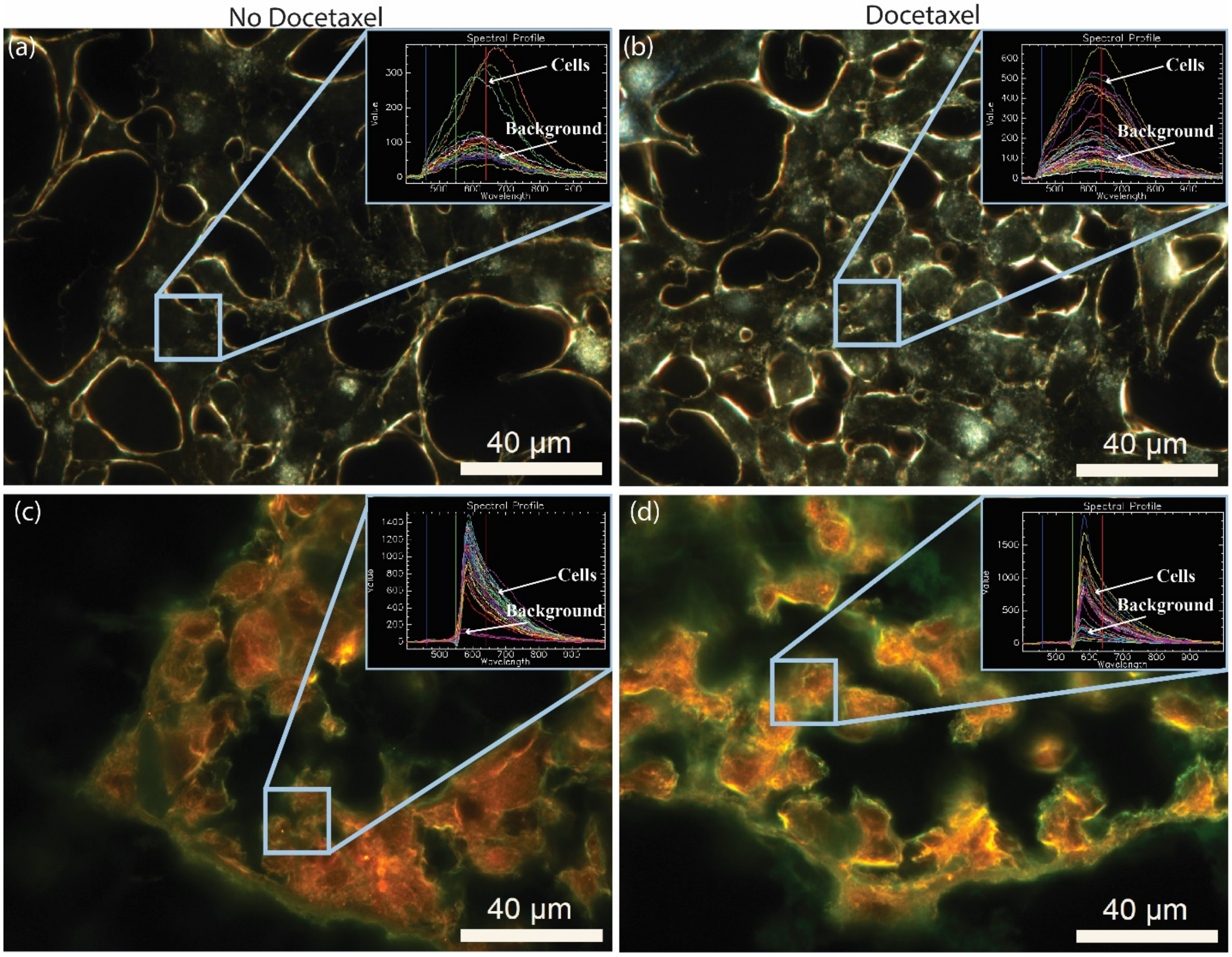

Figure 5. *Darkfield images of monolayer and spheroids.* (a,b) Darkfield images of a monolayer of LNCaP (a) without and (b) with docetaxel. (c,d) Darkfield images of 10 µm sections of LNCaP spheroids (c) without and (d) with docetaxel. Inset is hyper spectral spectrum of cells. Scale bar is 40 µm.

Supplementary Figure 6

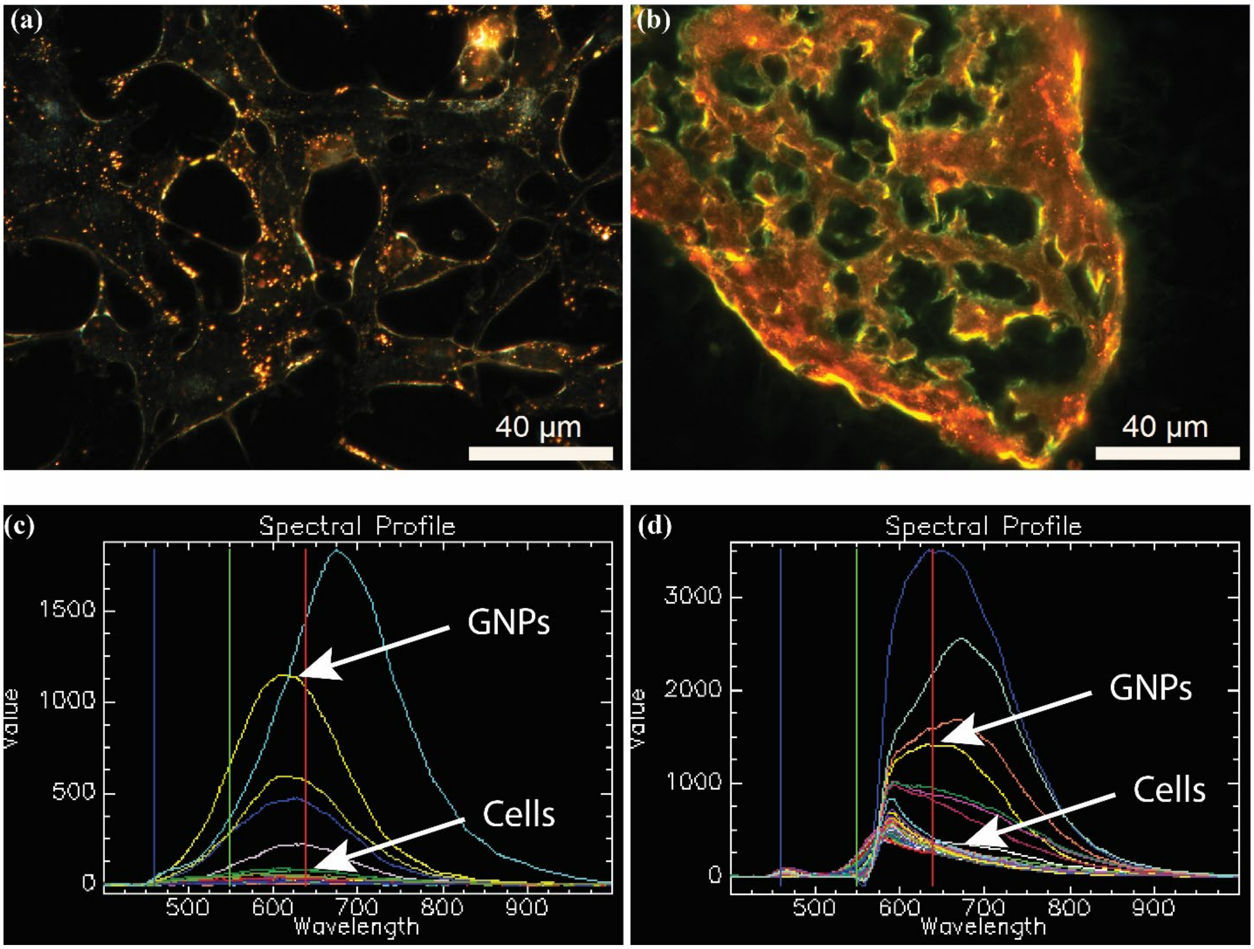

Figure 6. *Gold nanoparticle uptake in two-dimensional and three-dimensional models.* (a,b) Darkfield images of a (a) monolayer of LNCaP cells and (b) 10 µm section of a LNCaP spheroid treated with docetaxel and gold nanoparticles. Scale bar is 40 µm. (c,d) Hyper spectrum taken from hyper spectral images of the (c) monolayer of LNCaP and (d) LNCaP spheroid.

Supplementary Figure 7

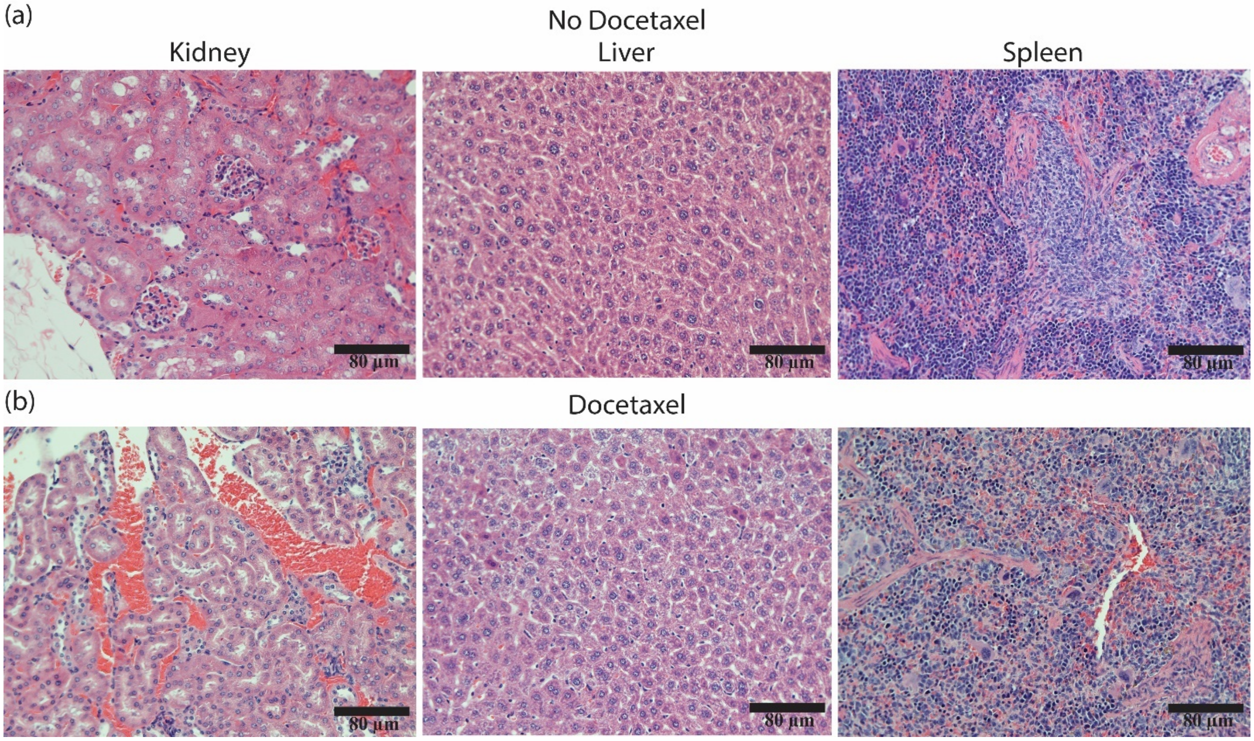

***Figure** 7. Hematoxylin and eosin-stained images of mouse tissue.* (a) Stained sections of 4 µm sections of the kidney, liver, and spleen without docetaxel. (b) Stained sections of 4 µm sections the kidney, Liver, and spleen with docetaxel after 24 hrs. Scale bar is 80 µum

Supplementary Figure 8

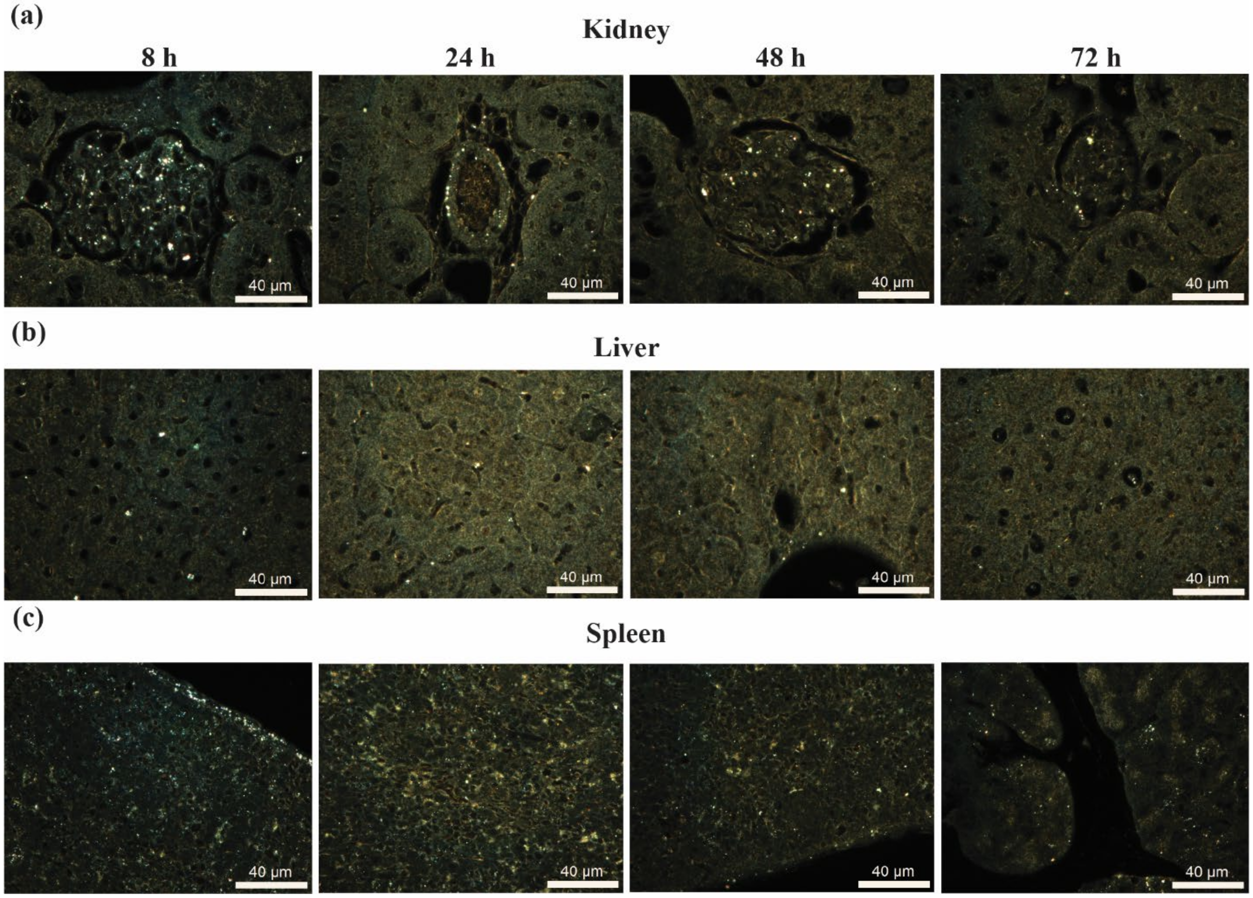

Figure 8. *Gold nanoparticles in mouse tissue with no docetaxel.* (a-c) Darkfield images of gold nanoparticles in a (a) kidney, (b) liver, and (c) spleen after 8, 24, 48, and 72 hrs of treatment. Scale bar is 40 µm.

Supplementary Figure 9

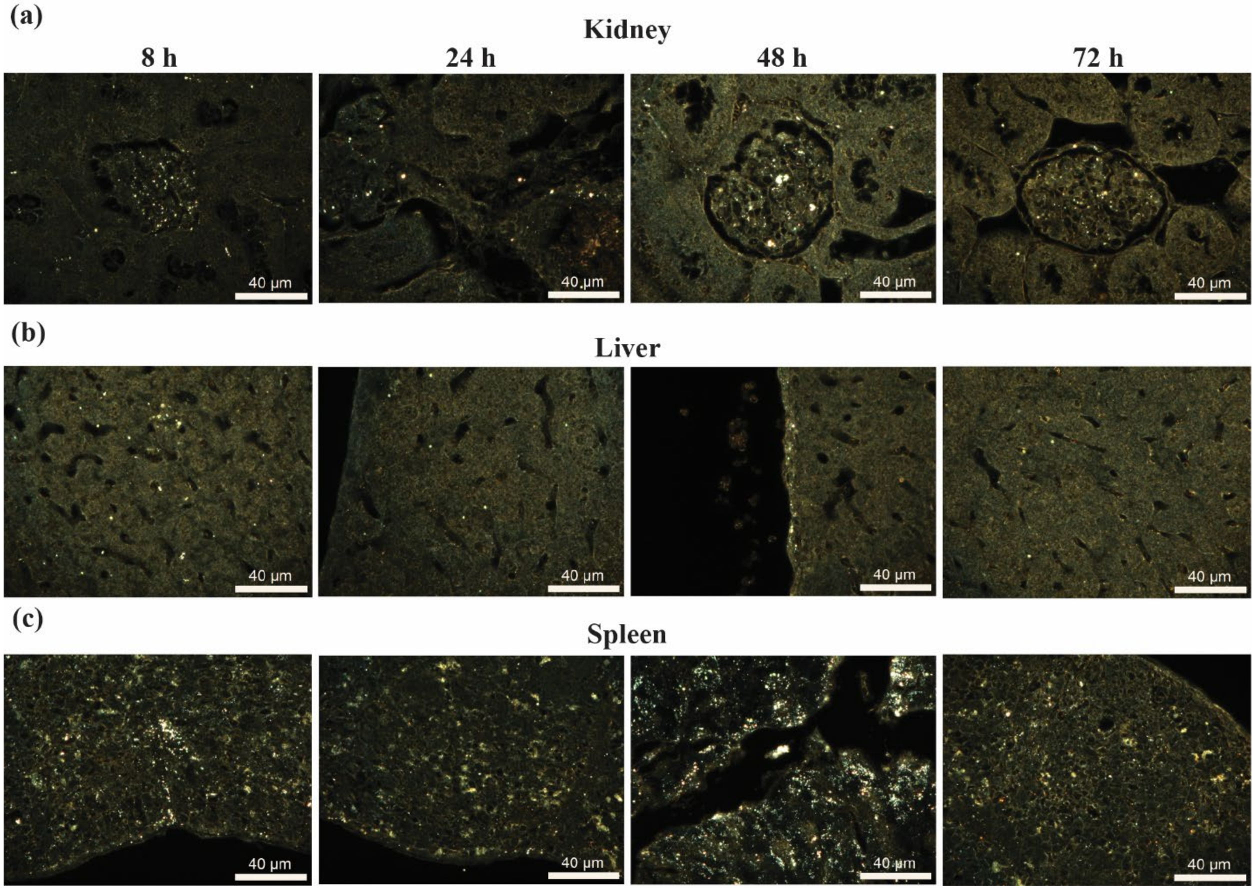

Figure 9. *Gold nanoparticles in mouse tissue with docetaxel.* (a-c) Darkfield images of gold nanoparticles in a (a) kidney, (b) liver, and (c) spleen treated with docetaxel after 8, 24, 48, and 72 hrs. Scale bar is 40 µm.
